# ﻿Two new species of *Stenoloba* Staudinger, 1892 from China (Lepidoptera, Noctuidae, Bryophilinae)

**DOI:** 10.3897/zookeys.1228.140176

**Published:** 2025-02-21

**Authors:** Jian Li, Chao Zhang, Hui-Lin Han, Vladimir S. Kononenko

**Affiliations:** 1 College of Forestry, Northeast Forestry University, Harbin 150040, China; 2 Simianshan Forest Resource Service Center, Jiangjin District, Chongqing, 402296, China; 3 Northeast Forestry University, Ministry of Education, Key Laboratory of Sustainable Forest Ecosystem Management, Harbin 150040, China; 4 Northeast Asia Biodiversity Research Center, Northeast Forestry University, Harbin 150040, China; 5 Laboratory of Entomology, Federal Scientific Center of the East Asia Terrestrial Biodiversity, Far Eastern Branch, Russian Academy of Sciences, Vladivostok-22, 690022, Russia

**Keywords:** *Basiviridis* species-group, hyperdiverse, morphology, moth, *nigrabasalis* species-group, taxonomy

## Abstract

Two new species of the moth genus *Stenoloba* Staudinger, 1892, *S.zhaotonga***sp. nov.** and *S.oculibasis***sp. nov.**, are described from China. The former includes photos of both female and male genitalia, while the latter only includes photos of male genitalia.

## ﻿Introduction

*Stenoloba* Staudinger, 1892 (type species *Dichagyrisjankowskii* Oberthür, 1884) represents a genus exclusively found in East Asia within the subfamily Bryophilinae, as established by Staudinger (1892). In the early 19^th^ century, several species belonging to this genus were described by various scholars (Moore 1888; Leech 1889; Hampson 1910; Warren 1914; Mell 1943; Draudt 1950). Currently, it is recognized as a large and relatively well-studied group, acknowledged as one of the most diverse within the subfamily Bryophilinae. Its distribution extends from India to Indonesia and includes regions such as China, the Korean Peninsula, Japan, and the southern part of the Russian Far East.

The taxonomic position of this genus has undergone several revisions over the years. Earlier classifications placed it within the subfamily Acontiinae [Acontianae] or Sarrothripinae (Hampson 1910; Warren 1914; Mell 1943; Draudt 1950). It was not until Sugi (1970) conducted a revision of the genus in Japan that *Stenoloba* was officially assigned to the subfamily Bryophilinae. Since the beginning of this century, a significant global revision of *Stenoloba* has been carried out by Kononenko and Ronkay (2000, 2001). Subsequently, the genus *Stenoloba* has demonstrated a sporadic pattern of development. Notably, significant contributions to the description and revision of new species within the genus from China (including Taiwan Island) and other regions of Southeast Asia, have been progressively undertaken by Chen (1999); Yoshimoto (1992, 1994); Kononenko and Ronkay (2000, 2001); Ronkay (2001); Sohn and Han (2005); Han and Lü (2007); Han and Kononenko (2009, 2015, 2018, 2021); Han and Kononenko et al (2020); Holloway (2009); Behounek and Kononenko (2010); Han et al. (2011); Pekarsky (2011); Sohn and Tzuoo (2012); Pekarsky and Saldaitis (2013); Pekarsky et al. (2013); Pekarsky (2018, 2019); and Saldaitis and Volynkin (2020). To date, a total of 103 species and 22 species groups of *Stenoloba* have been recognised. This genus exhibits amazing diversity in China, with 69 species classified into 19 species groups recorded here.

In the course of the inventory of Chinese Noctuoidea fauna, we investigated the diversity of *Stenoloba* in the southeastern part of China. Extensive material on the genus has been collected. Among them, two new species of *Stenoloba* from two species groups are described below.

## ﻿Material and methods

Specimens for this study were collected from the southern and southwestern regions of China using light traps. Adult specimens were prepared according to the standard Lepidoptera wing-spreading method. Dissection and slide preparation of the genitalia followed the techniques outlined by Kononenko and Han (2007). Adult images were captured using a Nikon D700 camera, while genitalia images were obtained with an Olympus BX51 microscope and the Qcapture Pro system. Image processing was conducted using Helicon Focus v. 7.0 and Adobe Photoshop 2023. The type specimens of the new species are deposited in the collection of Northeast Forestry University, Harbin, China.

### ﻿Abbreviations used

**NEFU** Northeast Forestry University, Harbin, China

**HT** holotype

**PT** paratype

**HHL** slide made by Hui-Lin Han

**LJ** slide made by Jian Li

## ﻿Taxonomic account

### ﻿Genus Stenoloba

Taxon classificationAnimaliaLepidopteraNoctuidae

Staudinger, 1892

EAAF74F4-02FE-53ED-8D8E-E2701F8484D5

Stenoloba Staudinger, 1892, in Romanoff, *Mémoires sur les Lépidoptéres* 6: 381. Type-species: Dichagyrisjankowskii Oberthür, 1884, *Etudes d’Entomologie* 10: 28, pl. 3: 5, by monotypy. Syntypes: [Russia, Primorye terr.] Sidemi (BMNH).
= Neothripa Hampson, 1894, *Fauna British India* (*Moths*) 2: 366, 382. Type species: Neothripapunctistigma Hampson, 1894, ibidem 2: 382, by original designation. Type(s): India: [Punjab] Simla (BMNH).
= Lepidopyrga Warren, 1914,Novitates zoologicae 21: 405. Type-species: Stenolobaviridimicta Hampson, 1910, Catalogue of the Lepidoptera Phalaenae in the British Museum 10: 369, pl. 159: 31, by original designation. Holotype: [India] Assam, Khasis (BMNH).
= Conicochyta Hampson, 1918,Novitates zoologicae 25: 137. Type-species: Chytonisolivacea Wileman, 1914, *Entomologist* 47: 165, by original designation. Holotype: [Taiwan] Formosa: Rantaizan (BMNH).

### ﻿ Stenoloba zhaotonga

Taxon classificationAnimaliaLepidopteraNoctuidae

Li, Han & Kononenko sp. nov.

E2A435A5-AC2E-5137-87B2-BC6A54684DE0

https://zoobank.org/5A48B7A5-EC48-4A24-B3CA-0AED98FCCABA

**Figures 1–6.**
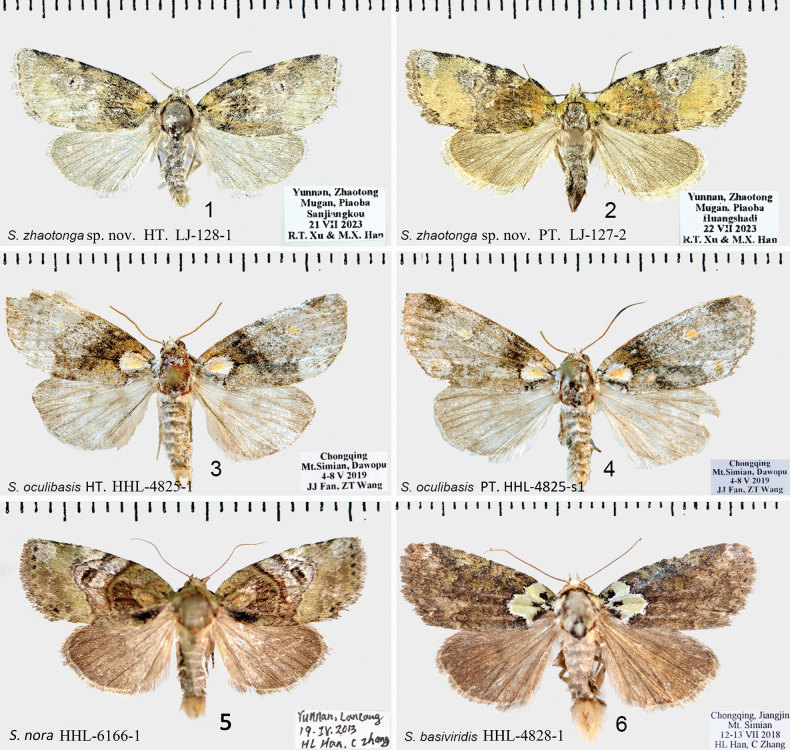
*Stenoloba* spp., adults. **1***S.zhaotonga* sp. nov., male, HT, slide LJ-128-1 **2***S.zhaotonga* sp. nov., female, PT, slide LJ-127-2 **3***S.oculibasis* sp. nov., male, HT, slide HHL-4825-1 **4***S.oculibasis*, sp. nov., male, PT, slide HHL-4825-s1 **5***S.nora*, male, slide HHL-6166-1 **6***S.basiviridis*, male, slide HHL-4828-1 [All materials from the collection of NEFU].

**Figures 7–10.**
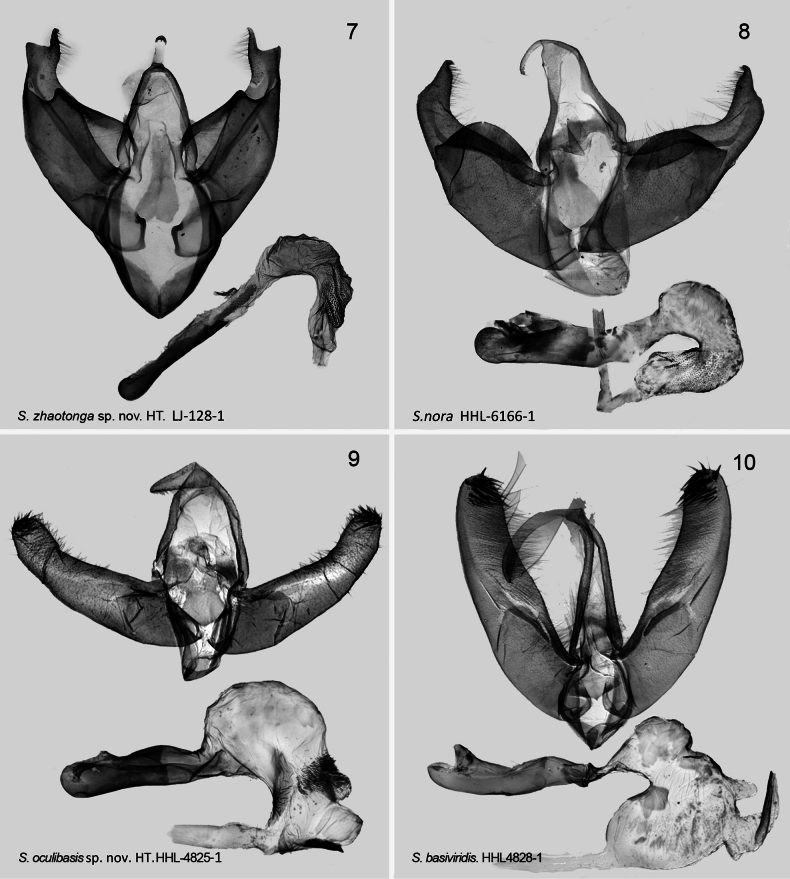
*Stenoloba* spp., male genitalia. **7***S.zhaotonga* sp. nov., HT, slide LJ-128-1 **8***S.nora*, slide HHL-6166-1 **9***S.oculibasis* sp. nov., HT, slide HHL-4825-1 **10***S.basiviridis*, male, slide HHL-4828-1 [All materials from the collection of NEFU].

**Figures 11, 12.**
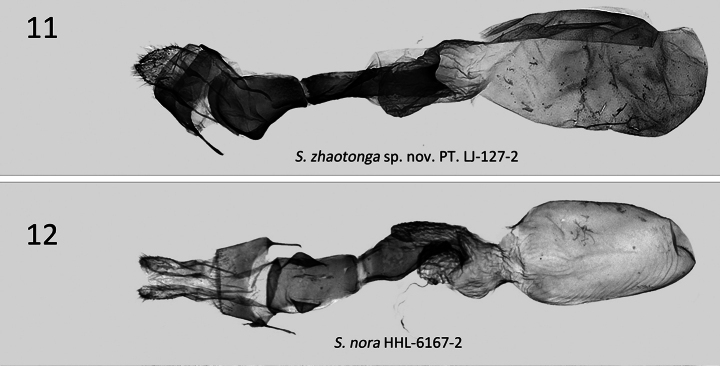
*Stenoloba* spp., female genitalia. **11***S.zhaotonga* sp. nov., PT, slide LJ-127-2 **12***S.nora*, slide HHL-6167-2 [all materials from coll. NEFU].

**Figure 13.**
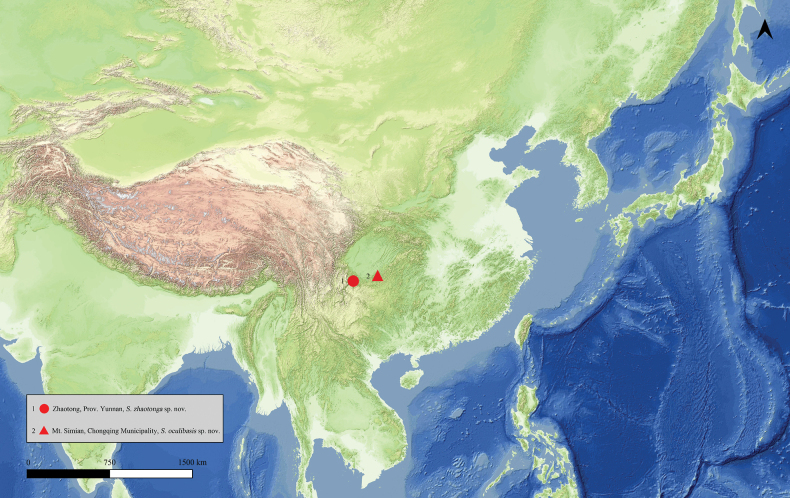
Map of collecting sites. **1** Zhaotong, Prov. Yunnan, *S.zhaotonga* sp. nov. **2** Mt. Simian, Chongqing Municipality, *S.oculibasis* sp. nov.

Figs 1 , 2 , 7 , 11 , 13

#### Type material.

***Holotype***: • 1♂, China, Prov. Yunnan, Zhaotong, Huangshadi, 22 July 2023, RT. Xu, MX. Han leg., slide LJ-128-1, coll. NEFU. ***Paratype***: • 1♀, China, Prov. Yunnan, Zhaotong, Sanjiangkou, 21 July 2023, RT. Xu, MX. Han leg., slide LJ-127-2, coll. NEFU.

#### Diagnosis.

The new species belongs to the *S.nigrabasalis* species-group. This group includes six species: *S.nigrabasalis* Chang, 1991, *S.ochribasis* Kononenko & Ronkay, 2001, *S.nora* Kononenko & Ronkay, 2001 (Figs 5, 8 12), *S.uncata* Han & Kononenko, 2018, *S.herbacea* Saldaitis & Volynkin, 2020 and *S.zhaotonga* Li, Han & Kononenko, sp. nov.

*Stenolobazhaotonga* sp. nov. shares several important characteristics with other species that are typical of the *S.nigrabasalis* species-group. The structure of the male genitalia reveals that the uncus is usually shorter; both sacculus and costa exhibit different shapes and are asymmetrical. The valve is relatively wide from the base to the middle, displaying strong hardening, with narrowing commences at the cucullus, where the outer edge is nearly straight; the costa features a protrusion resembling a finger or hill shape; additionally, the cucullus generally possesses a protruding structure that extends outward and includes cornua of varying lengths; finally, the juxta is large and either diamond-shaped or lingual.

In contrast to all species in the *S.nigrabasalis* species-group, *S.zhaotonga* is very similar to *S.nora*. In terms of adult features, *S.zhaotonga* has the head and thorax brown-yellow (in *S.nora* ochreous-green); forewing is overall brown-yellow (in *S.nora* more greyish); the basal area is yellow, with some filamentous dark brown stripes (in *S.nora* darkened, with a conspicuous wide blackish streak); reniform stigma is preceded by some irregular black spots (in *S.nora* dark spot behind reniform small or missing); and costal margin has dense dark brown stripes (in *S.nora* sparse). For male genitalia, uncus of the new species is clavate, relatively wide, short and flattened (in *S.nora* long and thin); juxta is elongate, shovel-like (in *S.nora* fusiform); apex of valva thin, straight, pointed, with a rectangular subapical process and both sides are densely covered with hairs (in *S.nora* cucullus has a small hook-like process); sacculus with elongated hill-like extension on outer margin, straighter (in *S.nora* more rounded); vesica sickle-shaped, dorsal side has cornuti and a granulation area, and cornuti are densely distributed, with cornute longer (in *S.nora* broadly tubular, recurved, ends have cornuti and granulation areas, cornute shorter). For the female genitalia, apophysis anteriores longer and much thicker (in *S.nora* shorter and thinner); tubular part of corpus bursae relatively long (in *S.nora* shorter).

#### Description.

***Adult*** (Figs 1, 2). Wingspan 24–27 mm. Head brown-yellow, with dense olive scales. Antennae filiform both in male and female. Labial palps sickle-shaped. Thorax brown-yellow, mixed with white. Abdomen brown-yellow with grayish-white scales. Ground colour of forewings brown-yellow, scattered with smoky gray and coffee tones. Costal and inner margins almost parallel. Wing veins visible; milky white between vein R_2_ and costal margin, with wavy brown markings. Apex slightly rounded, with gray spots; basal spots grayish-white; basal line blurred, jagged, tan; subbasal line sepia; antemedial line brown; medial area with irregular gray brown spots; reniform stigma brown, preceded by some irregular black spots, periphery with grayish-white radial stripes; postmedial line wavy, brown; subterminal line serrated, grayish-brown; terminal area with broken grayish-brown spots; fringe long, brown. Hindwing smoky gray, deeper in colour towards termen; outer margin decorated with grayish-brown fringe and brown discal spot.

***Male genitalia*** (Fig. 7). Uncus wide, short, mallet-shaped, about 1/3 length of tegumen; tegumen narrow, V-shaped, about 1.5 times shorter than vinculum; saccus V-shaped, strongly sclerotized; valva slightly asymmetric with mid of costal margin near straight and wide base, gradually narrowing to cucullus; cucullus bifurcated and extends outward vertically, forms conical protrusion, approximately same length as uncus; sacculus wide, sclerotised, with elongated hill-like extension on outer margin, blunted apically, strongly sclerotised; juxta elongate, shovel-like, extends posteriorly, approximately as long as tegumen. Aedeagus slender, almost straight, tubular; caecum slightly enlarged, strongly sclerotised in carinal plate; vesica sickle-shaped, covered in medial part with scobinate area in distal part, dorsal side has cornuti field and granulation area, and cornuti densely distributed.

***Female genitalia*** (Fig. 11). Papillae anales broad and conical; apophysis posteriors longer than apophysis anteriores, blunt, slightly extended proximally, anterior apophysis thicker, shorter; antrum large, deep, funnel-like; ductus bursae long, strongly sclerotized, with membranous ring in joining with ductus bursae; corpus bursae membranous, elongated sack-like, with strongly sclerotised appendix bursa at junction with ductus bursae.

#### Bionomics.

This species is known in Southwest China only from its type locality in Zhaotong, Prov. Yunnan, where it was collected in the mountainous regions at an altitude of about 1700 m. Both a male and a female specimen were collected in July.

#### Distribution.

(Fig. 13) Southwest China (Prov. Yunnan).

#### Etymology.

The species name refers to its collection site, Zhaotong area in Prov. Yunnan.

### ﻿ Stenoloba oculibasis

Taxon classificationAnimaliaLepidopteraNoctuidae

Li, Zhang, Han & Kononenko sp. nov.

801D4387-C0A3-58BD-A1AB-F0519D080EFA

https://zoobank.org/C49C097A-5327-46AB-BBCC-0D016A2F9D94

Figs 3 , 4 , 9 , 13

#### Type material.

***Holotype***: • 1♂, China, Chongqing Municipality, Mt. Simian, 4–8 May 2019, JJ. Fan, ZT Wang leg., slide HHL-4825-1, coll. NEFU. ***Paratype***: • 1♂, China, Chongqing Municipality, Mt. Simian, 4–8 May 2019, JJ. Fan, ZT Wang leg., slide HHL-4825-s1, coll. NEFU.

#### Diagnosis.

The new species belongs to the *S.basiviridis* species-group. This group includes 10 species: *S.basiviridis* Draudt, 1950 (Figs 6, 10), *S.assimilis* (Warren, 1909), *S.assimilina* Han & Kononenko, 2018, *S.gaoligonga* Han & Kononenko, 2018, *S.domina* Kononenko & Ronkay, 2000, *S.dominula* Kononenko & Ronkay, 2000, *S.siamensis* Behounek & Kononenko, 2010, *S.mossy* Behounek & Kononenko, 2010, *S.lampra* Kononenko & Ronkay, 2000 and *S.oculibasis* Li, Zhang, Han & Kononenko, sp. nov.

In comparison to several other species within the *S.basiviridis* species-group, *S.oculibasis* exhibits several distinctive characteristics, specifically: the uncus is generally elongated; the valva is long and rod-like, nearly straight, with a base that maintains approximately equal width only at its terminal end. The cucullus is rounded and features dense, long, and strongly sclerotized (or hardened) cornua. The juxta is typically spindle-shaped. Furthermore, the terminal vesica of the aedeagus possesses a long, spiky basal plate associated with the ductus ejaculatorius.

From the perspective of wing surface characteristics, *S.oculibasis* is different from other species in this species-group. In the base area of the forewing, there exists a large oval spot. The center of this spot is orange-yellow, surrounded by gray-white. The middle part of the wing is darker and brown, while it turns to gray-white in the outer edge area. An orange-yellow reniform stigma is behind the submedial line. The male genitalia of *S.oculibasis* have similarities with both *S.assimilina* and *S.basiviridis*. It differs from *S.assimilina* by the shorter and tapered uncus and the missing strong ventro-subapical spine in the apical part of the valva [in *S.assimilina* (Han and Kononenko 2018, fig. 43) spine is present and the uncus short, flattened, rather massive, but not triangulate]. Compared with *S.basiviridis*, in *S.oculibasis* sp. nov. the uncus is short, pyramidal, sharp (in *S.basiviridis* long rod-like, tip fine); the vesica is generally kidney-shaped, with a wide base, oval shape, and a narrow middle and cornuti field is with intensive cornuti on the dorsal side (in *S.basiviridis* the vesica armed with one large spine-like cornutus).

#### Description.

***Adult*** (Figs 3, 4). Wingspan 26–27 mm. Forewing overall grayish-white with ochre tint. Head grayish-white. Thorax grayish-white, mixed with taupe. Abdomen gray, mixed with brown. Forewing basal area with grayish-white scales, there is an oval spot at the base, orange yellow in the middle, gray white around, and black brown on the outer edge; basal line brown; subbasal line distinct, russet; antemedial line tan; antemedial area darker, brownish-yellow; median line and postmedial line tan; reniform stigma yellow surrounded by light grayish-white; postmedial area with longitudinal stripes along veins; apex rounded, with irregular tan apical patch; tornal patch orange; terminal line serrated, brown; fringe trim, brown. Hindwing, light coloured, grayish-white; discal spot brown. Forewing underside colour grayish-white, with different shades, scattered brown stripes, and wing veins clearly visible.

***Male genitalia*** (Fig. 9). Uncus relatively short, tapered, with wide base, pointed apically; tegument U-shaped; saccus V-shaped; valva long, almost equal in width from base to apex, slightly convex from base, sclerotised in apical half; sacculus wide, extending to about 1/3 length of valva, cucullus smooth, circularly arched, apex of cucullus armed with thorns and surrounded by long hair-like seta; juxta approximately pentagonal, extending upwards, sclerotised. Aedeagus straighter; caecum round; vesica reniform, cornuti field has dense cornutus, ventral area wrinkled and sclerotised, dorsally armed with large, wide, nail-like cornutus, and terminal has a long spiky basal plate of ductus ejaculatorius.

***Female genitalia*.** Unknown.

#### Bionomics.

The species is known only from its type locality in Southwest Chongqing Municipality, where it occurs in mountains at an altitude of 1100 m. Both male specimens were collected in May.

#### Distribution.

(Fig. 13) Southwest China (Chongqing Municipality).

#### Etymology.

The species name “*oculibasis*” refers to one of the two main distinguishing characters: the presence of the large white with a creamy centre rounded spot in the basal part of the forewing.

## Supplementary Material

XML Treatment for Stenoloba

XML Treatment for Stenoloba zhaotonga

XML Treatment for Stenoloba oculibasis

## ﻿Additional information

### Author ORCIDs

Jian Li https://orcid.org/0009-0002-4428-0617

Chao Zhang https://orcid.org/0000-0001-6069-1059

Hui-Lin Han https://orcid.org/0000-0002-2045-6182

Vladimir Kononenko https://orcid.org/0000-0001-6103-4800

### Data availability

All of the data that support the findings of this study are available in the main text.
